# Pharmacological Melanocortin 5 Receptor Activation Attenuates Glomerular Injury and Proteinuria in Rats With Puromycin Aminonucleoside Nephrosis

**DOI:** 10.3389/fphys.2022.887641

**Published:** 2022-06-01

**Authors:** Bohan Chen, Zubia Alam, Yan Ge, Lance Dworkin, Rujun Gong

**Affiliations:** ^1^ Division of Nephrology, Department of Medicine, University of Toledo Medical Center, Toledo, OH, United States; ^2^ The Center for Diabetes and Endocrine Research, University of Toledo, Toledo, OH, United States; ^3^ Center for Hypertension and Precision Medicine, University of Toledo Medical Center, Toledo, OH, United States; ^4^ Department of Physiology and Pharmacology, University of Toledo College of Medicine and Life Sciences, Toledo, OH, United States

**Keywords:** glomerular pathobiology, melanocortin 5 receptor, podocytopathy, GSK3β (glycogen synthase kinase 3β), puromycin aminonucleoside nephrosis

## Abstract

Clinical evidence indicates that the melanocortin peptide ACTH is effective in inducing remission of nephrotic glomerulopathies like minimal change disease (MCD) and focal segmental glomerulosclerosis (FSGS), including those resistant to steroids. This suggests that a steroid-independent melancortinergic mechanism may contribute. However, the type of melanocortin receptor (MCR) that conveys this beneficial effect as well as the underlying mechanisms remain controversial. Burgeoning evidence suggests that MC5R is expressed in glomeruli and may be involved in glomerular pathobiology. This study aims to test the effectiveness of a novel highly selective MC5R agonist (MC5R-A) in puromycin aminonucleoside (PAN) nephrosis. Upon PAN injury, rats developed evident proteinuria on day 5, denoting an established nephrotic glomerulopathy. Following vehicle treatment, proteinuria continued to persist on day 14 with prominent histologic signs of podocytopathy, marked by ultrastructural glomerular lesions, including extensive podocyte foot process effacement. Concomitantly, there was loss of podocyte homeostatic markers, such as synaptopodin and podocin, and *de novo* expression of the podocyte injury marker desmin. Treatment with MC5R-A attenuated urine protein excretion and mitigated the loss of podocyte marker proteins, resulting in improved podocyte ultrastructural changes. *In vitro* in cultured podocytes, MC5R-A prevented the PAN-induced disruption of actin cytoskeleton integrity and apoptosis. MC5R-A treatment in PAN-injured podocytes also reinstated inhibitory phosphorylation and thus averted hyperactivity of GSK3β, a convergent point of multiple podocytopathic pathways. Collectively, pharmacologic activation of MC5R by using the highly selective small-molecule agonist is likely a promising therapeutic strategy to improve proteinuria and glomerular injury in protenuric nephropathies.

## Introduction

The past three decades have witnessed major advances in understanding the cellular and molecular mechanisms of proteinuric glomerulopathies. Regardless of the initial trigger for disease, dysfunction or injury of glomerular podocytes, a key constituent of the glomerular filtration barrier that governs the glomerular permselectivity of proteins, mainly accounts for the pathogenesis of proteinuria in most nephrotic glomerulopathies (Daehn and Duffield, 2021). Nevertheless, refractory nephrotic syndrome continues to be a challenge for clinical practice and treatment. There is a pressing need to identify novel therapeutic targets and develop new therapeutic modalities to induce remission in proteinuric kidney disease. As one of the first FDA-approved medications for treating nephrosis, adrenocorticotropic hormone (ACTH) was widely used in the 1950s but was later replaced by synthetic corticosteroids (Adams et al., 1962). In addition to being a critical mediator of the hypothalamic-pituitary-adrenal (HPA) axis, the pituitary neuropeptide ACTH is also a key member of the melanocortin hormone family (Gong, 2011). Recent clinical evidence indicates that ACTH is indeed effective in inducing remission of nephrotic glomerular diseases like minimal change disease (MCD) and focal segmental glomerulosclerosis (FSGS) (Fernandez et al., 2001; Chakraborty et al., 2020), even those resistant to steroids. This suggests that a steroidogenic-independent melancortinergic mechanism may contribute (Gong, 2014). The melanocortin system consists of 5 receptors, namely melanocortin 1 receptor (MC1R) to MC5R (Cone, 2006). Different MCRs are expressed in a tissue-dependent pattern and can exert different physiologic activities and biologic functions (Gantz and Fong, 2003). However, the type of melanocortin receptor (MCR) that conveys this renoprotective effect of ACTH as well as the underlying mechanisms remain controversial.

**FIGURE 1:**
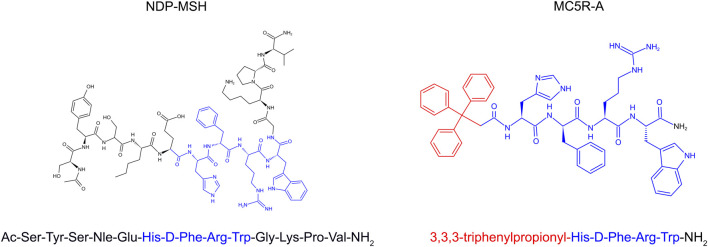
The 2-D view of chemical structures of synthetic melanocortin peptidomimetics, NDP-MSH and MC5R-A. The melanocortin core tetrapeptide, His-D-Phe-Arg-Trp is marked in blue color. The triphenylpropionyl group of MC5R-A is marked in red color.

A myriad of published studies have described the expression of all five types of MCR in the kidney and their involvement in the pathobiology of kidney diseases. Recent evidence suggests that MC5R is expressed in human glomeruli and the change in its expression is associated with glomerular diseases, such as FSGS and membranous nephropathy (Bergwall et al., 2018). In particular, MC5R expression is evident in human podocytes (Da Sacco et al., 2013). MC5R expression has also been described in murine glomeruli and, similarly, its change was associated with glomerular injury in murine models of diabetic kidney disease (GIPR^dn^-transgenic mice) or glomerular hypertrophy and sclerosis (bGH-transgenic mice) (Blutke, 2008). More recently, single cell-RNA sequencing indicated that MC5R expression is detected in glomerular podocytes (Wang et al., 2020). Previously, our group demonstrated that MC5R expression was probed by immunoblotting in rat glomeruli (Si et al., 2013). However, despite these findings, the role of MC5R in the pathogenesis of glomerular disease remains elusive. It is yet to be determined if therapeutic targeting of MC5R could modify the disease progression of proteinuric glomerulopathy. To address this issue, this study employed the rat model of puromycin aminonucleoside (PAN) nephrosis, a well-characterized model of nephrotic glomerulopathy, and tested the effectiveness of synthetic melanocortin peptidomimetics with MC5R activating activities, including the pan MCR agonist [Nle^4^, DPhe^7^]-α-melanocyte-stimulating hormone (NDP-MSH) and a novel highly selective MC5R agonist (MC5R-A). MC5R-A was generated by N-terminal modification of the melanocortin core tetrapeptide His-D-Phe-Arg-Trp-NH2 with an aromatic group (Figure 1A), resulting in a triphenylpropionyl melanocortin analog with 100-fold selective agonist activity on MC5R as reported previously (Holder et al., 2003).

## Materials and Methods

### Animal Studies

Animal studies were approved by the Institutional Animal Care and Use Committee (IACUC) of the University of Toledo, and they conform to US Department of Agriculture regulations and the National Institutes of Health guidelines for humane care and use of laboratory animals.

To induce the rat model of nephrotic glomerulopathy, male Sprague-Dawley rats at the age of 8 weeks received a single tail vein injection of puromycin aminonucleoside (PAN, 80 mg/kg, Adooq Bioscience LLC, Irvine, CA, United States). As control, rats were injected with saline. Five days after the PAN injection, rats were given daily subcutaneous injections with equal doses (0.7 μmol/kg/d) of MC5R-A (3,3,3-triphenylpropionyl-His-D-Phe-Arg-Trp-NH2), NDP-MSH (custom-made peptide from GL Biochem Ltd., Boston, MA, United States) or vehicle until animals were euthanized on day 14. Upon euthanasia, kidneys and urine were collected for further examination.

### Urine Protein Analysis

Urine protein concentration was measured using the Bradford protein assay kit (Thermo Fisher Scientific, Waltham, MA, United States). All results were corrected for the urine creatinine concentration measured by creatinine assay (BioAssay System, Hayward, CA) using the Cytation 5 multi-mode microplate reader (BioTek Instruments, Winooski, VT, United States).

### Transmission Electron Microscopy

For transmission electron microscopy, kidney cortical tissues were cut into small pieces (1 mm^3^), fixed with 2.5% glutaraldehyde (Electron Microscopy Sciences, Hatfield, PA, United States), and were processed by standard procedures. The grid sections were visualized using Talos L120C transmission electron microscope (Thermo Fisher Scientific). To determine the average foot process width, the liner size of the foot processes that touch the glomerular basement membrane was measured as previously described by ImageJ (Souza et al., 2016) (Version 1.52a National Institutes of Health, Bethesda, MD, United States).

### Isolation of Glomeruli

Glomeruli were isolated by a conventional sieving method as described previously (Rush et al., 2018). Briefly, following euthanasia and transection of inferior vena cava above the renal veins, rat kidneys were perfused with ice-cold PBS *via* the left ventricular cannulation or the abdominal aorta cannulation until the kidneys had blanched. After perfusion, the left kidneys were quickly removed, and the cortices were minced into less than 1 mm in size. The minced cortices were gentally processed through graded sieving by application of pressure to the flat end of syringe plunger. The glomeruli trapped on the 75 μm sieve were collected for further examination.

### Cell Culture

The enriched glomeruli were plated on collagen type I-coated dishes at 37°C in RPMI 1640 medium with 10% FBS, 100 U/ml penicillin, and 100 μg/ml streptomycin (Life Technologies) in a humidified incubator with 5% CO_2_. Podocytes at passage 1 or 2 were characterized by the expression of podocyte-specific markers and used in subsequent experiments. Cells were treated with PAN (100 μg/ml) for 48 h after 30-min pretreatment with MC5R-A (10^−7^ mol/L) or NDP-MSH (10^−7^ mol/L), whose dose was adopted according to previous studies (Carniglia et al., 2016).

### Western Immunoblot

Isolated glomeruli were homogenized, and cells were lysed in radioimmunoprecipitation assay buffer supplemented with a protease inhibitor cocktail (Thermo Fisher Scientific). Samples were subjected to Western immunoblot analysis as described before (Li et al., 2016). The anti-synaptopodin, Wilm’s Tumor-1 (WT-1) and podocin antibodies were purchased from Santa Cruz Biochemistry. The anti-desmin, phospho-glycogen synthase kinase 3β (p-GSK3β), GSK3β and β-tubulin antibodies were acquired from Cell signaling Technology. For immunoblot analysis, bands were scanned, and the integrated pixel density was determined using the ImageJ analysis program.

### Immunofluorescence Staining

Cryosections of kidney specimens or cultured cells were fixed, permeabilized, and stained with antibodies against indicated molecules followed by staining with a secondary antibody conjugated with Alexa Fluor 488 or 594 or stained with rhodamine-conjugated phalloidin (Invitrogen, IL, United States) followed by counterstaining with 4,6-diamidino-2-phenylindole (DAPI, Abcam). Images were documented by using the Nikon Eclipse Ni-U microscope (Nikon, Tokyo, Japan) and the Leica TCS SP5 multiphoton laser scanning confocal microscope (Leica Microsystems Inc., Buffalo Grove, IL, United States).

### TUNEL Staining

Apoptotic cell death in cell cultures was detected by using the TUNEL kit (Roche Molecular Biochemicals, Mannheim, Germany) according to the manufacturer’s instruction. After TUNEL staining, cells were counterstained with propidium iodide (PI, Thermo Fisher Scientific) and visualized by using the Nikon Eclipse Ni-U microscope (Nikon, Tokyo, Japan).

### Wound Healing Assay

Confluent monolayers of primary podocyte cultures were scraped with a 10 μL pipette 30 min after different treatments. Phase-contrast micrographs were obtained at 0 and 24 h after scratching by using EVOS FL microscope (Thermo Fisher Scientific). The wound areas at 0 and 24 h were analyzed using ImageJ and data were expressed as the ratio of wound closure as previously reported (Bao et al., 2015).

### Statistical Analyses

All the data are expressed as mean ± SD. Statistical analysis of the data from multiple groups was performed by one-way ANOVA tests followed by Tukey’s tests. Statistical analyses were performed using GraphPad Prism 8.0 software (GraphPad Software, San Diego, CA, United States). *p* < 0.05 was considered statistically significant.

## Results

### Delayed Treatment With the Selective MC5R Agonist Attenuates Proteinuria in Rats With PAN Nephrosis and Improves the Structural Integrity of Glomeruli

**FIGURE 2:**
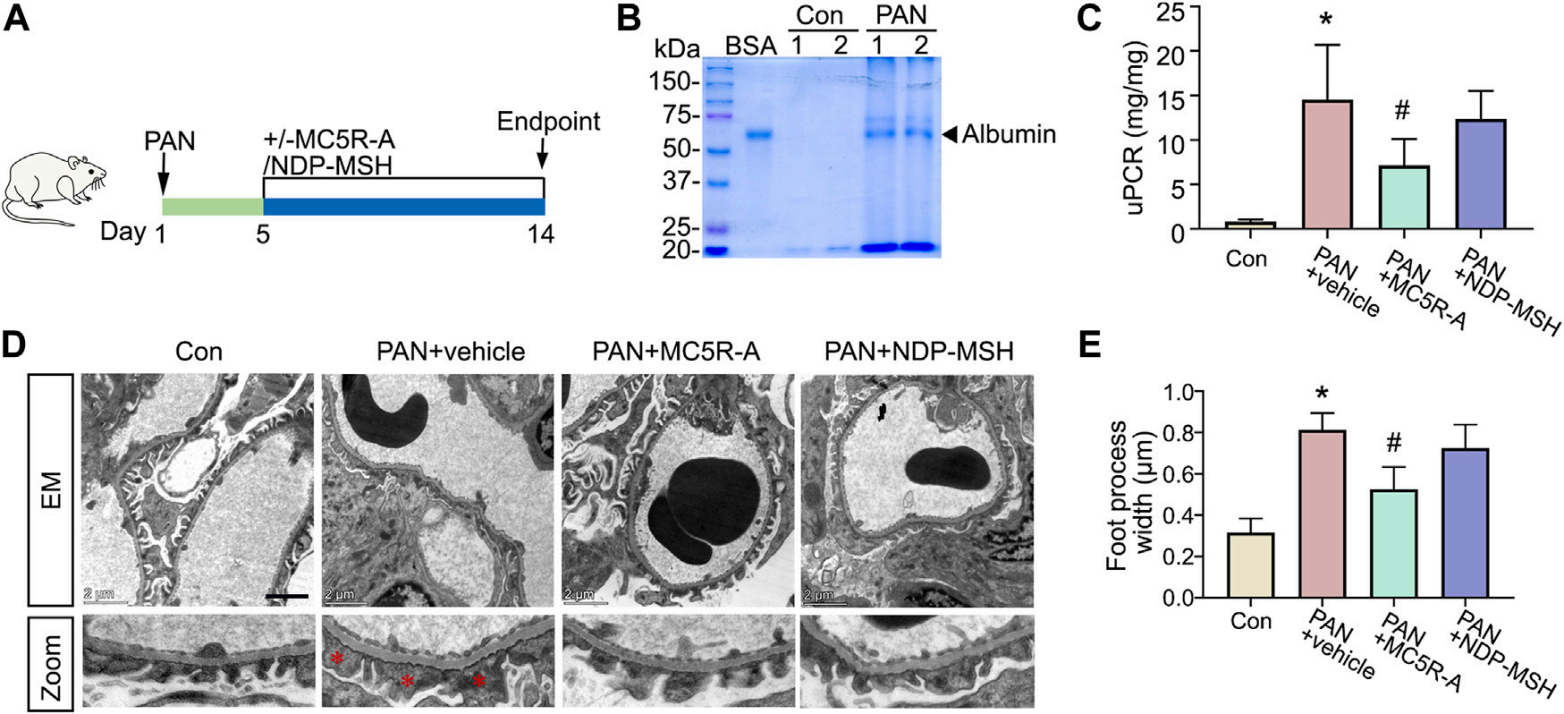
MC5R agonist attenuates proteinuria and podocyte injury in the rat model of PAN nephrosis. **(A)** Schematic diagram depicts the animal study design. On day 1, rats received a tail-vein injection of PAN or saline as normal controls (Con). Five days after the PAN injection, rats received daily subcutaneous injections of MC5R-A, NDP-MSH or vehicle until day 14, when rats were euthanized. **(B)** An aliquot of urine collected on day 5 was subjected to SDS-PAGE followed by Coomassie brilliant blue staining. Bovine serum albumin (BSA, 3 μg) served as a standard control. **(C)** Urine protein and creatinine levels were assayed, and urinary protein excretion was expressed as urinary protein to creatinine ratio (uPCR). ∗*p* < 0.05 *versus* Con group; ^#^ *p* < 0.05 *versus* PAN + vehicle group (*n* = 6). **(D)** Representative electron microscopic images of glomerular ultrastructures (Scale bar = 2 μm); The asterisks indicate foot process effacement. **(E)** Quantification of podocyte foot process width; ^∗^ *p* < 0.05 *versus* control group; ^#^ *p* < 0.05 *versus* PAN + vehicle group (*n* = 4).

Upon PAN injury, rats developed heavy proteinuria on day 5 as indicated by urine protein electrophoresis, exhibiting an established nephrotic glomerulopathy (Figure 2B). Following vehicle treatment, proteinuria continued to persist on day 14, as quantitated by urine protein to creatinine ratios (Figure 2C). The proteinuria was accompanied by prominent histologic signs of podocytopathy (Figures 2D,E), marked by ultrastructural glomerular lesions, including extensive podocyte foot processes effacement. Daily treatment with MC5R-A markedly reduced proteinuria and showed a considerable improvement in podocyte foot process effacement (Figure 2). Daily treatment with NDP-MSH also exerted a beneficial action that marginally attenuated proteinuria in spite of no statistical significance. This was associated with a trend toward improvement in glomerular ultrastructure.

### Podocyte Loss and Dedifferentiation in Rats With PAN Nephrosis Are Mitigated by the Selective MC5R Agonist

**FIGURE 3:**
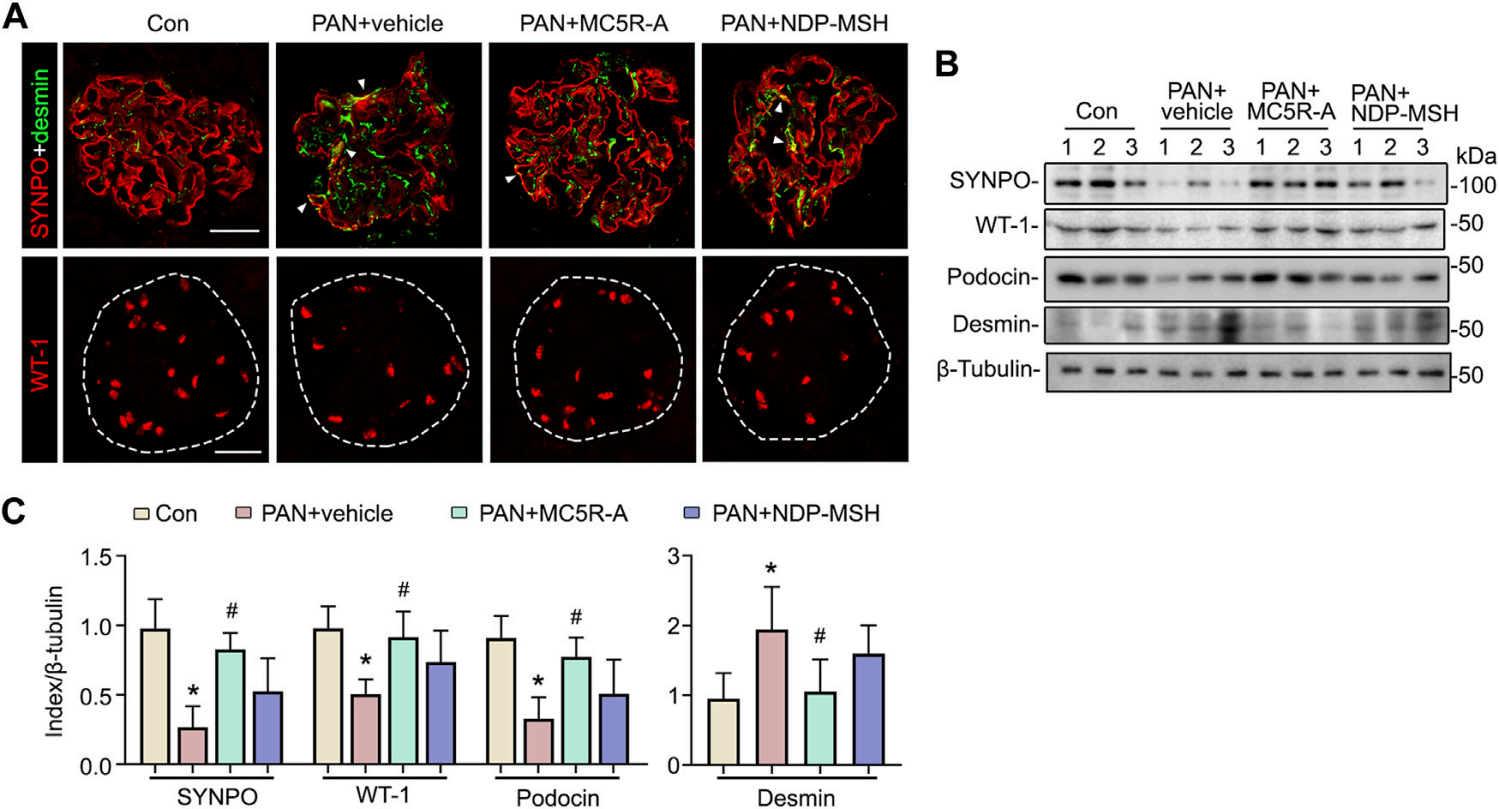
MC5R agonist exerts a protective effect on glomerular injury and podocytopathy in PAN nephrosis rats. **(A)** Animals were treated as elaborated in Figure 2. Cryosections of kidney tissues procured from various groups were subjected to immunofluorescence staining for the podocyte-specific markers, including synaptopodin (SYNPO) and WT-1, as well as for desmin, a marker of podocyte injury. Representative micrographs are shown (Scale bar = 50 μm). White arrowheads indicate yellow signals positive for both SYNPO (red) and desmin (green), denoting *de novo* expression of desmin in podocytes. **(B)** Isolated glomeruli were processed for immunoblot analysis for SYNPO, WT-1, podocin, desmin, as well as β-tubulin, which served as the loading control. **(C)** Quantification of the indicated protein expression levels by densitometric analyses of immunoblots, expressed as relative levels normalized to β-tubulin. ^∗^ *p* < 0.05 *versus* the same index expression of control (Con) group; ^#^ *p* < 0.05 *versus* the same index expression of PAN + vehicle group (*n* = 6).

Glomerular injury in rats with PAN nephrosis has been associated with podocyte degeneration (Luimula et al., 2002) featured by podocytopenia, loss of podocyte homeostatic markers and dedifferentiation. To examine podocyte changes, kidney specimens were processed for fluorescent immunohistochemistry staining for podocyte marker proteins synaptopodin, WT-1, and desmin, a muscle-specific intermediate filament and a podocyte injury marker. Shown in Figure 3A, in vehicle-treated rats with PAN nephrosis, glomerular expression of synaptopodin was substantially repressed after PAN injury with *de novo* expression of desmin in podocytes, as indicated by increased positivity of dual staining for synaptopodin and desmin, suggesting podocyte dedifferentiation. This was accompanied by evident podocytopenia as shown by reduced numbers of WT-1-positive podocytes in glomeruli. All these histologic signs of podocyte degeneration were significantly mitigated after MC5R-A treatment. The morphologic findings were further corroborated by immunoblot analysis of isolated glomeruli for synaptopodin, podocin, desmin and WT-1 (Figures 3B,C). NDP-MSH treatment also conferred a protective effect, but only resulted in a trend toward improvement in glomerular expression of synaptopodin, desmin and WT-1 with no statistical difference.

### Activation of MC5R in Rat Podocytes Protects Against Actin Cytoskeleton Disruption Elicited by PAN

**FIGURE 4:**
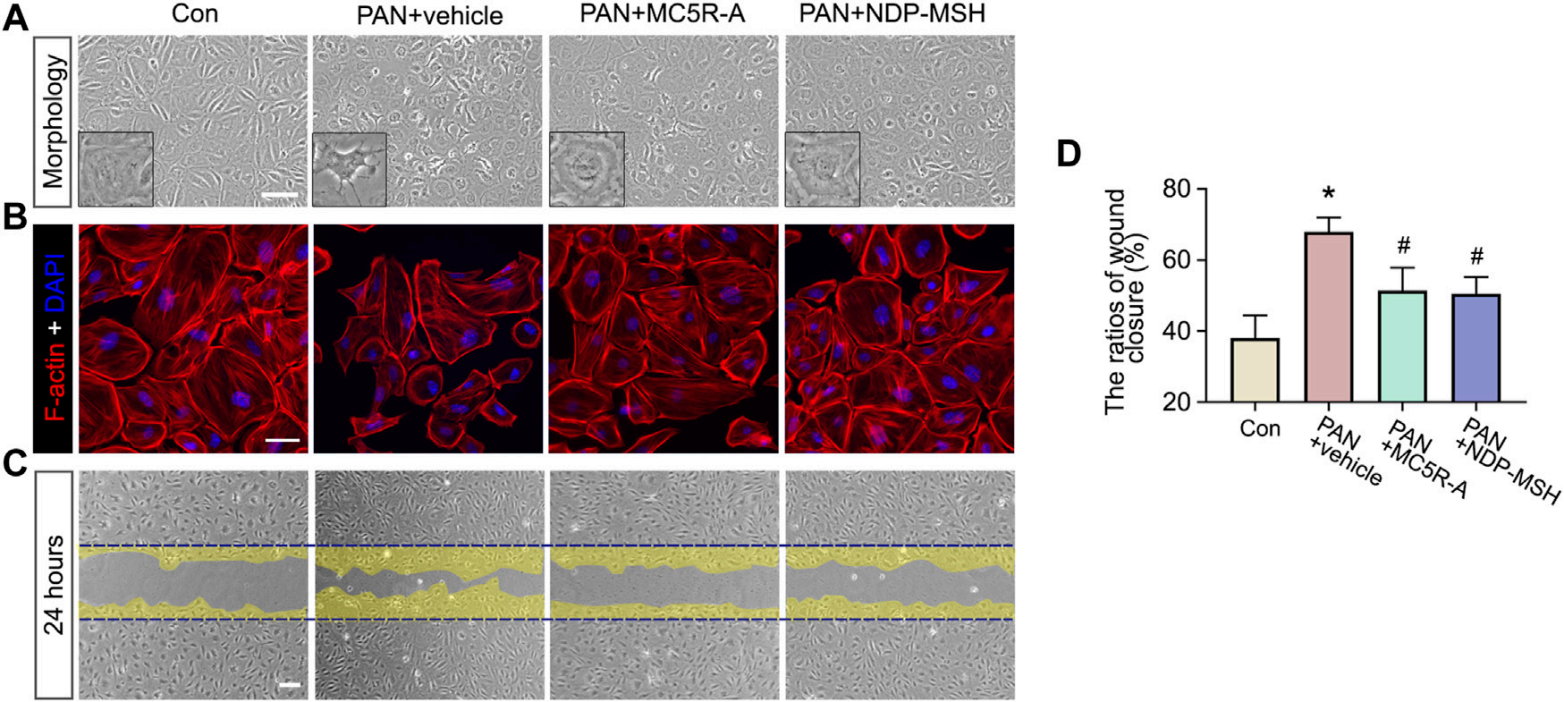
MC5R agonism protects podocytes against PAN-induced disruption of cytoskeleton integrity and podocyte hypermotility. Podocytes were treated with PAN after pretreated with MC5R-A, NDP-MSH or vehicle for 30 min. **(A)** Representative phase-contrast micrographs show podocyte shape changes (Scale bar = 100 μm). **(B)** Cells with different treatment were fixed and subjected to F-actin staining followed by DAPI counterstaining (Scale bar = 50 μm). **(C)** Confluent monolayers of podocytes were scraped with a 10 μL pipette 30 min after different treatments. Representative micrographs taken at 24 h were shown. The cell migration areas were marked as yellow (Scale bar = 100 μm). **(D)** Computerized morphometric analysis of wounding areas following the indicated treatments. Data were expressed as the ratios of wound closure. ^∗^ *p* < 0.05 *versus* control (Con) group; ^#^ *p* < 0.05 *versus* PAN + vehicle group (*n* = 3).

To determine if the beneficial effect of MC5R-A in PAN stems from a possible direct effect on podocytes, primary podocyte cultures were prepared from isolated rat glomeruli and subjected to PAN injury in the presence or absence of MC5R-A or NDP-MSH treatment. Shown in Figures 4A,B, PAN stimulation elicited podocyte injury, marked by podocyte shape changes from large, flat, and arborized cell shapes to shrinkage, reduced arborization and aster like shapes (Figure 4A), in concert with disruption of the actin cytoskeleton rich in phalloidin-labeled filamentous actin stress fibers (Figure 4B). The cytoskeleton derangement upon PAN injury was associated with podocyte hypermotility, as shown by an accelerated closure of the gap between the invading fronts of podocyte sheets based on a traditional cell migration assay for assessing cellular motility (Figures 4C,D). MC5R-A or NDP-MSH co-treatment mitigated the disruption of the actin cytoskeleton integrity and significantly attenuated podocyte motility, suggesting that the injurious effect of PAN was lessened by MC5R-A or NDP-MSH co-treatment.

### MC5R Agonist is Sufficient to Prevent PAN-Induced Podocyte Apoptosis

**FIGURE 5:**
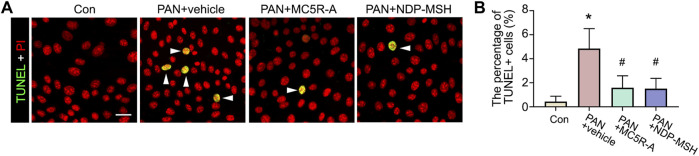
Podocyte apoptosis elicited by PAN is attenuated by the MC5R agonist. Cells were treated as described in Figure 4. **(A)** Cells with different treatments were fixed and subjected to TUNEL staining counterstained with PI (Scale bar = 50 μm); The arrowheads indicate TUNEL positive cells. **(B)** The percentage of TUNEL positive (+) cells per microscopic field. ^∗^ *p* < 0.05 *versus* control (Con) group; ^#^ *p* < 0.05 *versus* PAN + vehicle group (*n* = 3).

PAN stimulation could cause podocyte death, and this may contribute to podocytopenia (Fernandez et al., 2001; Min et al., 2018). Indeed, in PAN-injured podocytes, apoptotic cellular death was noted, as shown by TUNEL staining (Figure 5A). MC5R-A or NDP-MSH co-treatment obviously reduced the number of TUNEL positive cells. The morphologic finding was further corroborated by the absolute count of TUNEL positive cells as a percent of the total amount of cells per microscopic field (Figure 5B).

### MC5R Agonism Counteracts PAN‐Induced Hyperactivity of the Podocytopathic Mediator GSK3β in Podocytes

**FIGURE 6:**
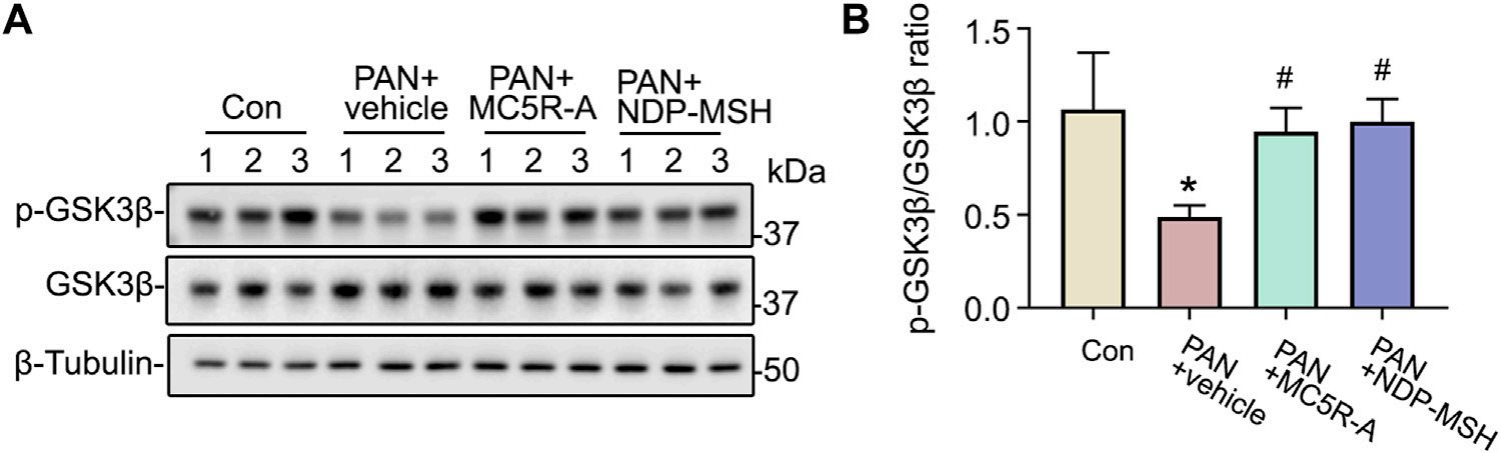
MC5R agonist treatment reinstates inhibitory phosphorylation of GSK3β. Cells were treated as elaborated in Figure 4. **(A)** Cells were lysed and processed for immunoblot analysis for p-GSK3β, GSK3β as well as β-tubulin, which served as the loading control. **(B)** Quantification of the p-GSK3β and GSK3β expression levels by densitometric analyses of immunoblots, expressed as relative levels normalized to GSK3β. ^∗^ *p* < 0.05 *versus* control (Con) group; ^#^ *p* < 0.05 *versus* PAN + vehicle group (*n* = 3).

Recent evidence suggests that some podocytopathic signaling mediators, such as GSK3β, are implicated in podocyte injury and glomerular disease (Li et al., 2016). To determine if the cytoprotective effect of MC5R-A or NDP-MSH is associated with any changes in GSK3β signaling, podocytes were collected and subjected to immunoblot analysis for p-GSK3β and GSK3β. Shown in Figure 6, PAN injury repressed the inhibitory phosphorylation of GSK3β and reduced the densitometric ratio of p-GSK3β to total GSK3β, denoting GSK3β hyperactivity. This effect was lessened by MC5R-A or NDP-MSH co-treatment (Figure 6B), suggesting that correction of the dysregulated GSK3β signaling may play a role in mediating the podocyte protective activity of MC5R-A or NDP-MSH.

## Discussion

Although glomerular expression of MC5R has been repeatedly described in rodents and humans, the role of MC5R in kidney disease is unknown. Our present study demonstrated that MC5R agonism is likely protective in the rat model of PAN nephrosis, as shown by the anti-proteinuric and glomerular protective effects.

MC5R was the last member of the MCR family to be cloned and characterized in the mid-1990s (Labbe et al., 1994). Its biological function has been mainly associated with sebogenesis, lacrimal secretion, release of sex pheromones and regulation of sexual behavior (Xu et al., 2020). More recent data suggest that MC5R activation may additionally exert a direct cellular protective action. For instance, in cultured cardiomyocytes, exposure to high ambient glucose elicited a 40% reduction in cell viability. This harmful effect of high glucose could be prevented by PG-901, a selective agonist of MC5R or by α-MSH, a pan MCR agonist. In consistency, *in vivo* studies in diabetic rats showed that PG-901 treatment also produced a cardiac protective effect (Trotta et al., 2018). Moreover, in mice with streptozotocin (STZ)-induced diabetic retinopathy, PG-901 protected against structural and microvascular changes in retinas (Rossi et al., 2016). Conversely, treatment with PG20N, a highly selective antagonist of MC5R exacerbated diabetic retinopathy (Rossi et al., 2016). Similar to the above findings, our study showed a podocyte protective activity of MC5R agonism. To this end, in cultured podocytes, co-treatment with MC5R-A protected against podocyte apoptosis elicited by PAN, denoting a cellular protection. Furthermore, the PAN-disrupted cytoskeleton integrity was mitigated by MC5R-A, associated with a correction of podocyte hypermotility as shown by cell migration assay. *In vivo*, MC5R-A treatment attenuated podocyte loss in rats with PAN nephrosis, again suggesting a mechanism of podocyte protection. How does MC5R signaling protect the cells? Our study suggests that the beneficial effect of MC5R-A in PAN-injured podocytes was associated with an averted hyperactivity of GSK3β, a convergent point of multiple podocytopathic pathways. Indeed, GSK3β hyperactivity has been implicated in podocyte death, cytoskeleton derangement and hypermotility in multiple injury models (Li et al., 2016). The inhibitory effect of MC5R activation on GSK3β is evidence of podocyte protection. It is unknown if GSK3β inhibition is directly caused by MC5R signaling or secondary to an improved podocyte homeostasis. But Trotta *et al* have similarly demonstrated that MC5R agonism increased the intracellular PI3K activity in cardiomyocytes, which was mediated by a decrease of the levels of the miRNA miR-133a (Trotta et al., 2018). GSK3β is a typical signaling mediator downstream of the PI3K-Akt pathway. It is conceivable that the potentiated PI3K activity after MC5R agonism would be associated with GSK3β inhibition. Thus, inhibition of GSK3β may be a common signaling event in different cell types following MC5R activation. This warrants further examination by furture studies.

Apart from a potential cytoprotective effect on glomerular podocytes, other mechanisms may also contribute to the renoprotective action of MC5R agonism. MC5R has been recently implicated in the modulation of immune response and inflammatory reaction (Jun et al., 2010; Lee and Taylor, 2013). Multiple immune cells, including T and B lymphocytes, mast cells, macrophages, antigen-presenting cells and others, have been shown to express MC5R (Taylor and Namba, 2001). In mice with experimental autoimmune uveoretinitis (EAU), a model of human autoimmune uveitis, Taylor et al. demonstrated that MC5R is involved in the protection of retina against the inflammatory damage and in the induction of ocular autoantigen responsive CD4^+^ regulatory T cells in the post EAU spleen (Taylor et al., 2006). Furthermore, in mice with STZ-induced diabetic retinopathy, intravitreal injection of the MC5R peptidomimetic agonist PG-901 attenuated, while MC5R antagonist PG20N augmented, retinal release of various inflammatory cytokines, such as IL-1α, IL-1β, IL-6, MIP-1α, MIP-2α, and MIP-3α (Rossi et al., 2016). The PAN nephrosis model involves not only glomerular cytotoxicity but also immune dysregulation. In support of this, TNF-α can directly trigger glomerular injury in rats (Taylor and Namba, 2001) and its production by monocytes collected from animals with PAN injury was found to be elevated (Bertani et al., 1989; Gómez-Chiarri et al., 1994). In addition, macrophage-derived foam cells are present in diseased glomeruli in the PAN models (Magil and Cohen, 1989). More importantly, PAN nephrosis could be markedly improved by classical immunosuppressants, such as cyclosporine (Kokui et al., 1992) and methylprednisolone (Fujiwara, 1984). Considering the possible immunomodulatory effect of MC5R activation, there is reason to hypothesize that MC5R-A may attenuate proteinuria and reduce glomerular injury in the PAN model indirectly *via* systemic immune regulation. In-depth studies are merited to validate this potential therapeutic mechanism.

If MC5R is a therapeutic target for proteinuric glomerulopathy, we question why the proteinuria-reducing efficacy of the potent pan-MCR agonist NDP-MSH seems to be less than that of MC5R-A? In fact, a similar finding was also made in a previous study in mice with Adriamycin nephropathy (ADN), in which treatment with α-MSH, a natural pan-MCR agonist that is much less potent and less stable than NDP-MSH, barely ameliorated albuminuria despite a trend toward improvement in podocyte foot process effacement (Lindskog Jonsson et al., 2014). In addition, in mice with LPS-induced podocytopathy, treatment with NDP-MSH, which is a more potent and more stable peptidomimetic of α-MSH, only resulted in a modest reduction in albuminuria (Qiao et al., 2016). As a pan-MCR agonist, NDP-MSH or α-MSH can activate all nonsteroidogenic MCRs including MC5R. It is unclear why this pan-MCR agonist seems less potent in reducing proteinuria in podocytotoxic models like the PAN nephrosis model or ADN model, but the following possibilities are consistent with our data or previous findings. First, there is evidence suggesting that NDP-MSH or α-MSH has a powerful hypertensive activity as a result of MC4R activation (Giuliani et al., 2007). Therefore, it is possible that the potential effect of NDP-MSH on systemic or renal hemodynamics may cause glomerular hyperperfusion and thereby counteract the anti-proteinuric action secondary to podocyte protection. Another explanation is that NDP-MSH or α-MSH, despite being claimed to be a pan-MCR agonist, does not have equal agonistic activities on all MCRs, but has the greatest activating activity on MC1R that is about 25 times greater than the MC5R agonistic activity (Winget et al., 2020). Thus, it is possible that at the dose of NDP-MSH used in our animals, MC1R rather than MC5R was fully activated. Due to unknown reasons, MC1R activation seems to be insufficient to protect against podocytopathy *in vivo* in rodent models like the ADN model (Lindskog Jonsson et al., 2014) or possibly in the PAN model here, although MC1R agonism did successfully protect against podocyte injury *in vitro* (Elvin et al., 2016; Bergwall et al., 2018). Further studies are absolutely needed to explore the mechanism underlying the descripancy in the efficacy of different melanocortin agonists in animal models of glomerular diseases.

Our study is not without limitations. Although MC5R-A has been claimed to be a highly selective MC5R agonist, it is derived from the pan-MCR agonist tetrapeptide His-D-Phe-Arg-Trp-NH2. As such, our study cannot rule out the possibility that some off-target effects or activation of other types of MCR may contribute to the observed beneficial action. To validate the findings here, transgenic animals with MC5R knockout or knockin would be the ideal tool to determine the exact role of MC5R in glomerular disease.

In summary, activation of MC5R by a highly selective peptidomimetic agonist ameliorated proteinuria and glomerular injury in rats with PAN nephrosis. This beneficial action was associated with a cytoprotective effect on podocyte injury that averted cytoskeleton disruption, hypermotility and apoptosis. Our findings suggest that MC5R may be a promising target for treating proteinuric glomerular diseases.

## Data Availability

The original contributions presented in the study are included in the article/Supplementary Material, further inquiries can be directed to the corresponding author.
